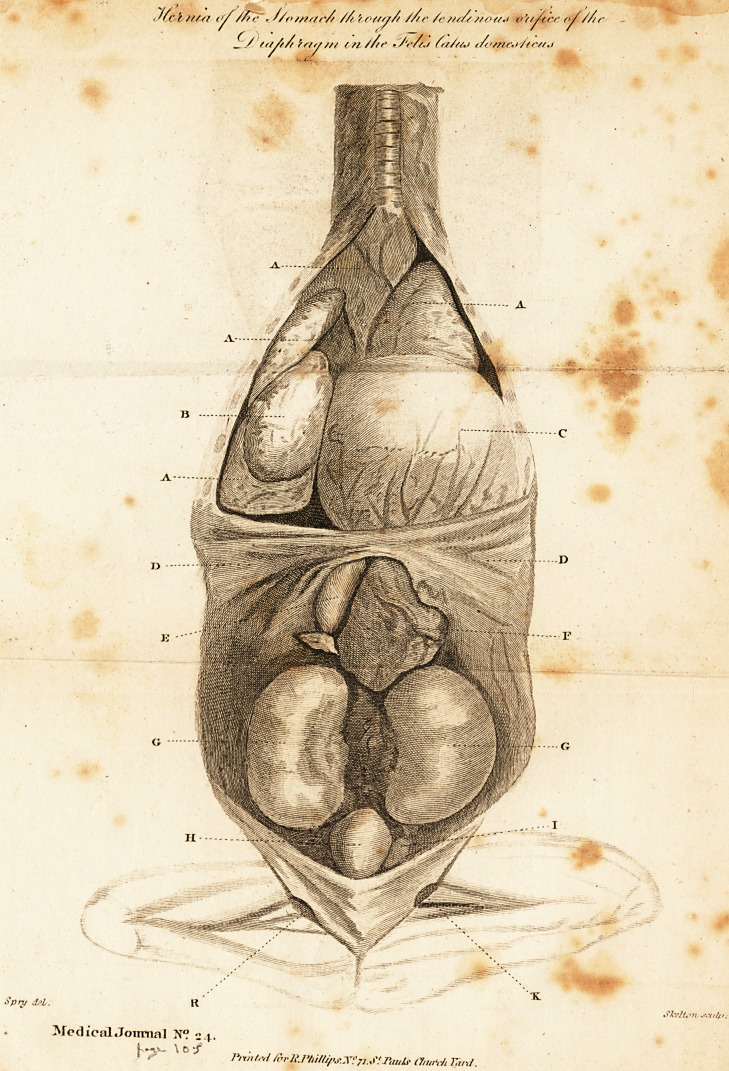# Mr. Spry's Case of Hernia

**Published:** 1801-02

**Authors:** James Hume Spry

**Affiliations:** Member of the Royal College of Surgeons, London. Aldersgate Street


### Medical and Physical Journal.

VOL. V.] FEBRUARY, 1801. [No. XXIV.

### *To the Editors of the Medical and Physical Journal.*

See Plate at begin of Vol

GENTLEMEN,

AS an extraordinary fact, perhaps you may be induced to give a place in your Journal to the following morbid dissection; the drawing is a correct representation of the disease, taken by myself from the preparation. Instances have occurred of hernia through the diaphragm in the human subject, but these instances are extremely rare; and when they have been discovered, a mal-conformation of the parts has at the same time been detected, which lusus naturæ was probably, in most cases, the primary cause of the accident. In the present instance, it did not appear upon dissection, that the diaphragm was malformed, or any of the adjacent parts; but the hernia appeared to have been produced by the common causes of hernia. As the erect position of the human body is peculiarly favourable to the production of the different species of hernia which pass through the ring of the external oblique muscle of the abdomen, and underneath that portion of its tendon, denominated the ligamentum poupartii, so likewise is the horizontal posture of different quadrupeds particularly favourable to the production of hernia of the diaphragm; but it does not appear that this morbid derangement has been detected by anatomists in the brute creation, at least, I am not acquainted with any one instance upon record; that it should not very often happen in the human subject, is not to be wondered at; indeed, so peculiar is the situation of the diaphragm, and so unfavourable is the erect position of the human body to its production, that, upon a cursory view of the subject, we might, with no small share of plausibility, as well as probability, on our side, pronounce that it never could happen, unless there was some very extraordinary mal-formation of the parts immediately concerned.

The drawing which I have now ſent you, has been ſhewn to ſeveral medical men, who have thought it worthy to be preſerved. If, Gentlemen, you ſhould be of a ſimilar opinion, it is much at your ſervice. I am, &c.

JAMES HUME SPRY,
Member of the Royal College of Surgeons, London.

*Aldersgate Street, December* 30, 1800.

---

The animal was obſerved to be in very great pain, and was ſeized ſuddenly, without having ſuffered any previous injury from external violence. It cried frequently, with a peculiar voice not to be deſcribed; and during reſpiration, (which was extremely difficult) it made a noiſe, which appeared to me, ſimilar to that which a child makes who labours under cynanche trachealis. Soon after its firſt ſeizure, it vomited very much; and upon attempting to put a little liquid into its mouth, the animal's tortures ſeemed to be very much increaſed: From the time of its ſeizure to its death, it was about two hours and a half, or three hours.

The ſtrangeneſs of the animal's complaint, induced me to diſſect it; and the following is a deſcription of the appearances which refer to the drawing, by which it will be much better underſtood. It is to be obſerved, that all the parts which were not abſolutely neceſſary to explain the diſeaſe, were removed, and omitted in the drawing.

AAAA. The Lungs.

B. The heart on the right ſide: the pericardium was ruptured, and the heart was forced from its natural ſituation by the ſtomach, which was exceedingly diſtended with air.

C. The Stomach; this viſcus occupied the major part of the thorax; and the lungs were forced up to the ſuperior part of the cheſt, and compreſſed in every direction by it.

DD. The diaphrgam.

E. The Duodenum.

F. A maſs of omentum, with the pancreas and ſpleen paſſing with the duodenum through the tendinous opening of the diaphragm.

GG. Renes.

H. Veſica urinaria.

I. Inteſtinum rectum.

KK. Opening of the external oblique muſcle of the abdomen, commonly called the abdominal ring.

## Figures and Tables

**Figure f1:**